# Drug–Drug Interactions in Elderly Patients with Potentially Inappropriate Medications in Primary Care, Nursing Home and Hospital Settings: A Systematic Review and a Preliminary Study

**DOI:** 10.3390/pharmaceutics13020266

**Published:** 2021-02-16

**Authors:** Mathilde Bories, Guillaume Bouzillé, Marc Cuggia, Pascal Le Corre

**Affiliations:** 1 Pôle Pharmacie, Service Hospitalo-Universitaire de Pharmacie, CHU de Rennes, 35033 Rennes, France; mathilde.bories@chu-rennes.fr; 2 Univ Rennes, CHU Rennes, INSERM, LTSI-UMR 1099, F-35000 Rennes, France; guillaume.bouzille@univ-rennes1.fr (G.B.); marc.cuggia@univ-rennes1.fr (M.C.); 3 Laboratoire de Biopharmacie et Pharmacie Clinique, Faculté de Pharmacie, Université de Rennes 1, 35043 Rennes, France; 4 Univ Rennes, CHU Rennes, Inserm, EHESP, Irset (Institut de recherche en santé, environnement et travail)-UMR_S 1085, F-35000 Rennes, France

**Keywords:** drug interactions, aged, potentially inappropriate medications, hospital, nursing home, primary care

## Abstract

Drug–drug interactions (DDI) occurring with potentially inappropriate medications (PIM) are additional risk factors that may increase the inappropriate character of PIM. The aim of this study was (1) to describe the prevalence and severity of DDI in patients with PIM and (2) to evaluate the DDI specifically regarding PIM. This systematic review is based on a search carried out on PubMed and Web-of-Science from inception to June 30, 2020. We extracted data of original studies that assessed the prevalence of both DDI and PIM in elderly patients in primary care, nursing home and hospital settings. Four hundred and forty unique studies were identified: 91 were included in the qualitative analysis and 66 were included in the quantitative analysis. The prevalence of PIM in primary care, nursing home and hospital were 19.1% (95% confidence intervals (CI): 15.1–23.0%), 29.7% (95% CI: 27.8–31.6%) and 44.6% (95% CI: 28.3–60.9%), respectively. Clinically significant severe risk-rated DDI averaged 28.9% (95% CI: 17.2–40.6), in a hospital setting; and were approximately 7-to-9 lower in primary care and nursing home, respectively. Surprisingly, only four of these studies investigated DDI involving specifically PIM. Hence, given the high prevalence of severe DDI in patients with PIM, further investigations should be carried out on DDI involving specifically PIM which may increase their inappropriate character, and the risk of adverse drug reactions.

## 1. Introduction

A drug–drug interaction (DDI) is usually defined as a clinically significant unintended modification in the exposure and/or response to a medication (i.e., victim) that occurs with the co-administration of another medication (i.e., perpetrator) [1]. Studies in the field of DDIs usually refer to a potential DDI, defined as the co-prescription of two medications known to interact, that may occur in exposed patients. DDIs can be categorized as either of pharmacokinetic origin (i.e., modification in exposure) or of pharmacodynamic origin (i.e., modification in response) [2]. A pharmacodynamic DDI results from the concomitant administration of medications that have the same sites of action, leading to additive, synergistic or antagonistic effects altering the drug effect with usually no apparent alteration in drug exposure. A pharmacokinetic DDI results from alterations in the processes involved in drug disposition when two drugs are co-administered. Basically, these DDI are due to either metabolic enzymes and/or transporters localized in membranes and tissues involved in absorption, distribution, metabolism or drug excretion, leading to apparent alteration in drug exposure.

Metabolism mediated by cytochrome P450 isoenzymes (principally CYP450 1A2, 2C9, 2C19, 2D6 and 3A4) is known to play a major part in the biotransformation of drugs in vivo, and in the interindividual variability in drug response. Drug transporters are categorized into two superfamilies: ATP binding cassette (ABC) transporters and solute carrier transporters (SLC) [3]. Among transporters, P-gp is the efflux transporter most commonly involved in DDI [4] The clinical consequences of drug interactions depend on the magnitude of their impact on the systemic exposure (i.e., area under the curve (AUC)). The intensity (strong, moderate or weak) of metabolic inhibition or induction is defined by the Food and Drug Administration (FDA) based on the impact on AUC [5]. Indeed, a strong inhibitor for a specific CYP is defined as an inhibitor that increases the AUC of a substrate for a more than 5-fold, or more than 80%, decrease in clearance [5]. For moderate and weak inhibitors, the increase is 2-fold to 5-fold, and 1.25-fold to 2-fold, respectively. An inhibition is a quite immediate phenomenon (occurring in the 24 h post dosing) while an induction requires the synthesis of new proteins (enzymes or transporters), reaching its maximum at around 7 to 10 days [5]. Thus, in clinical practice, we have to pay particular attention to inhibition mechanisms at either enzyme level or transporter level.

The risk of DDI is higher in elderly patients since they have many co-morbidities that exposed them to polymedications. The risk of DDI is potentially increased by age-related modifications in drug pharmacokinetics and pharmacodynamics [6]. Prevention of DDI in the elderly should be integrated in a medication action plan that not only assesses DDI but also drug–disease interactions, identification and reconsideration of high risk therapy and adjustment for organ elimination [7]. Care pathways of elderly people involve several settings from primary care to nursing home and hospital, with different complexity in the therapeutic management depending on the degree of severity of the diseases.

In a systematic review, DDI was identified as a significant cause of ADR-related hospitalizations with a median DDI prevalence rate of 22.2% and 8.9% for hospital admission and hospital visits, respectively [8]. In a prospective study (Effets indésirables des Médicaments: Incidence et Risque (EMIR) study, *n* = 2692 admissions), the incidence of hospital admissions related to ADRs was 3.6% [9] with 29.9% of these ADR-related hospitalizations resulting from DDI. 

Within elderly people exposed to polymedications, prescription of potentially inappropriate medications (PIMs) is an issue. PIMs correspond to medications with different characteristics: i.e., medications that are potentially inappropriate in most older adults, medications that are potentially inappropriate in older adults with certain conditions, medications that should be used with caution, potentially clinically important DDI to be avoided in older adults and medications that should be avoided or have their dosage reduced based on kidney function [10]. With regard to DDI, it should be kept in mind that Beers classification only considers a limited number of potentially clinically significant interactions. Hence, the prevalence of clinically significant interactions in elderly adults is obviously higher than estimated by Beers criteria [10]. Within all the DDI occurring in the elderly in general, DDI occurring specifically with PIM as victim drug is an issue since these DDI could enhance their inappropriate character. Indeed, the inhibition in drug metabolism or transporter-dependent elimination increases the systemic exposure of a PIM and may lead to increase in incidence and/or severity of ADRs and their consequences (i.e., re-hospitalization and/or mortality). Hence, the knowledge of the prevalence of DDI occurring specifically with PIM as victim drugs in elderly patients should be considered as a public health subject, and as of interest for the health professionals in drawing their attention to these specific DDI.

The aim of this study was to perform a systematic review in hospital, nursing home and primary care settings to evaluate the prevalence and severity of DDI occurring in elderly patients for whom PIM are prescribed, and then to evaluate the DDI involving specifically PIM.

## 2. Materials and Methods

This review was performed according to the Preferred Reporting Items for Systematic Reviews and meta-analyses (PRISMA) statement. The PRISMA check list [11] and PRISMA flow chart are available in Supplementary material 1 and 2.

### 2.1. Search Strategy

We conducted a systematic search of the published peer-reviewed literature in Pubmed and Web of Science databases. Duplicate manuscripts were removed after exporting references in Zotero reference management software (www.zotero.org (accessed on 13 February 2021)).

A literature search was conducted using Medical Subject Headings (MeSH) terms and text words around the topic of drug–drug interactions and potentially inappropriate medication use in older people.

The literature search was carried out from inception to June 1, 2020 without language restrictions, and non-English publications were translated prior to data extraction. Details of the full search strategy are available in Supplementary material 3.

### 2.2. Eligibility Criteria

Original studies reporting the prevalence of DDI and potentially inappropriate medications. 

Studies were considered eligible to be included in this review if they provided the age of involved patients. Looking at patient’s characteristics, most of the studies focused on patients older than 65 years old. However, two studies selected patients above 60 years old (Brazil and Pakistan). Our choice to include them in this review is based on the WHO definition of an older person in developing countries which has set the threshold at 60 years old for these countries [12]. The following exclusion criteria were applied: duplicate studies, letters, reviews.

### 2.3. Study Selection

Two authors (MB and PLC) independently assessed the publications for inclusion in the review. The titles and abstracts were screened to identify the potentially relevant studies aiming at determining the prevalence of DDI and of PIM in older patients. The full-text copies for the studies that apparently met the criteria as well as those for which an uncertainty existed were retrieved for an independent review by the two authors (MB and PLC). If the two authors did not reach agreement through discussion about a paper to be included, we consulted a third study author (MC) to resolve any disagreement.

### 2.4. Data Extraction and Synthesis

Two review authors (MB, PLC) independently extracted relevant data from full-text articles using a predetermined data extraction form. In case of disagreement, the resolution was obtained by discussion between the two review authors, and a third author (MC) was consulted if necessary. 

The following items were included in the extraction form: first author, year, country (ies) of origin, age of patients (mean, threshold if mean non available), study setting (primary care or medical institution), sample size, typology of patients (general population or specific disease), study style, source of data, software or tool used for checking prevalence of PIM and DDI, mean number of medications.

Data were collected from the publications directly as reported or indirectly (i.e., after calculation from the raw data). Any missing data were requested from the study authors.

### 2.5. Data Analyses

The prevalence of DDI and of PIM corresponds to the percentage of patients that had at least one DDI or one PIM. 

We decided to report in detail only the DDI considered as clinically significant; that is, DDI with major and moderate risk. There was a diversity of tools used to check the DDI, and the risk rating classification of DDI differed between some of them. Hence, we have carried out a mapping of the different DDI checker tools to integrate only DDI we considered as major and moderate risk according to the description that was reported by each tool.

We made the following comparisons:

(a) Prevalence of each parameter (PIM and DDI with severe and moderate risks) reported by the most used tools (using reported mean) without consideration of settings, in a first time. Then, we made the same comparison using computed weighted mean and 95% CI for the most used tools to evaluate potential differences between settings.
(b) Prevalence of PIM and DDI (with severe and moderate risks) in each setting (using computed weighted mean).
(c) Polymedication between settings (number of prescribed medications per patient reported as the mean).

### 2.6. Statistics

As a result of differences in the number of patients involved the studies we computed a weighted mean for each parameter taking into account the sample size. We used the *svymean* and *confint* functions from the R survey package to compute weighted means and 95% confidence intervals (CI95%) [13]. Statistical analyses were performed on data from articles focusing on the general population using Shapiro Wilk and Kruskal Wallis tests. For pairwise comparisons of the prevalence of PIM and DDI (with severe and moderate risks) in each setting, a false discovery rate correction for alpha risk was used. Statistical analysis was performed using R software (version 3.6.3). A *p*-value < 0.05 was considered significant.

### 2.7. Mapping of DDI Risk Classification

Since there are variations in the classification of DDI among DDI softwares, we made a mapping of the DDI risk classification based on the descriptions given by the providers. We retained and classified the DDI we considered major (e.g., contra-indicated/serious/life threatening) or moderate (e.g., use with caution).

### 2.8. Data Extraction from Hospital Clinical Data Center

In the course of this review, we noticed that very few studies were dealing with DDI involving specifically PIM so that we decided to perform a preliminary study within our university hospital clinical data warehouse (CDW) [14]. In order to estimate the prevalence of DDI specifically with PIM, we have chosen 6 PIM from different therapeutic areas (tramadol, apixaban, digoxine, clozapine, glimepiride and quetiapine) with a narrow therapeutic index that are metabolized and/or transported by the most common pathways involved in drug pharmacokinetics (CYP450 isoenzymes: 1A2, 2C9, 2C19, 2D6, 3A4–Transporter: P-gp). For each PIM (as substrate), we have selected some interacting medications known to lead to a DDI rated as moderate or major intensity. This led to a panel of 15 DDI whose prevalence were screened in electronic health records of elderly patients from our CDW (hospitalized in 2019).

## 3. Results

### 3.1. Study Selection

The literature search identified 212 studies in Pubmed and 298 in Web of Science databases leading to 440 studies after removal of duplicate studies. After titles and abstract screening, full-text articles were assessed for legibility. The flow chart is depicted in Supplementary Material 2.

### 3.2. Study Characteristics

Three study groups were created according to the study setting: nursing home (*n* = 20), primary care (*n* = 38) or hospital settings (*n* = 33). Among these 91 studies, 66 focused on general population and 25 on specific populations (population with specific medications or specific diseases, e.g., oncology, psychiatric disease, etc.).

Included studies were published from 1994 to 2019. Most of the studies from general population were conducted in Europe (*n* = 38) and North America (*n* = 13). However, other countries from Asia (*n* = 8) or South America (*n* = 7) have also published results. Further details on the characteristics of the studies and the tools used are available as Supplementary Material 4.

Table 1, Table 2 and Table 3 describe the characteristics of each study evaluated in this review for nursing home, primary care and hospital settings, respectively.

### 3.3. Mapping of DDI Checker

Our study showed that different DDI checker tools are used in different ways in risk rating DDIs (Table 1, Table 2 and Table 3). The mapping of severity classifications of the different tools that were used is presented in Table 4. Among DDI checker tools, Micromedex and Lexicomp were most frequently used (*n* = 17, 18% and *n* = 10, 10%. respectively). The Swedish Physicians’ Desk Reference tool was also frequently used, especially as a result of numerous studies (*n* = 9) performed in Sweden in primary care and nursing home settings. Based on these elements, comparison between studies is not straightforward as a result of difference in DDI detection.

### 3.4. Potentially Inappropriate Medications

We checked a potential difference between the tools used to identify PIMs by comparing Beers Criteria, STOPP/START, Swedish National Board of Health and Welfare, Fick list and NORGEP that were the most used tools (47%, 11%, 11%, 8% and 5%, respectively). The average prevalence reported by using these tools were 38.6%, 60.4%, 25.0%, 29.2% and 26%, respectively. Without consideration of settings, the distribution was not normal (*p* = 0.0018). We found a significant difference between the tools (*p* = 0.020) in their ability to detect PIMs.

Since Beers Criteria was the most used tool in the general population (*n* = 35, 47%), we made a comparison of the prevalence of PIM according to Beers between the settings. The prevalence of PIMs in primary care, nursing home and hospital were 26.1% (95% CI: 25.8–26.4), 20.1% (95% CI: 1.6–38.5) and 43.9% (95% CI: 23.2–64.5), respectively. Differences between the settings were found (*p* = 0.0062).

Without tool consideration, the weighted mean prevalence of PIM in primary care, nursing home and hospital was: 19.1% (95% CI: 15.1–23.0%), 29.7% (95% CI: 27.8–31.6%) and 44.6% (95% CI: 28.3–60.9%). The Kruskal–Wallis test used to compare the prevalence of PIM between settings showed significant differences (*p* = 0.0027). Using pairwise comparisons, significant differences were found between nursing homes and primary care (*p* = 0.0029) and between hospital and primary care (*p* = 0.0037) but not between nursing home and hospital (*p* = 0.4604).

The prevalence of PIM in specific populations of patients and in the different settings is presented in Table 1, Table 2 and Table 3. In the specific populations, given the diversity of diseases encountered (especially mental health diseases in nursing home and oncology in hospital settings), comparison between settings is not relevant. However, it should be noticed that PIM prevalence in the specific populations was higher than in the general populations except for hospital setting (35.0% in specific population vs. 44.6% in the general population).

### 3.5. Polymedication

Polymedication did not display a normal distribution (*p* = 0.023) and revealed no differences between settings (*p* = 0.6741). Indeed, the number of medications per patient in primary care, nursing home and hospital was 7.07, 7.02 and 7.44, respectively.

### 3.6. Drug–Drug Interactions

Our mapping of the different risk rating classification provided by the checker tools allowed us to report only clinically relevant DDI, i.e., considered as major and moderate risk (Table 4).

We checked for a potential difference between the tools used to identify DDI by comparing Micromedex, Lexicomp, DRUID, Swedish Physician’s Desk reference and Intercheck that were the most used tools to identify DDI (in 20%, 7%, 7%, 13% and 6% of the studies, respectively). The average prevalence of DDI reported by using these tools were 19.9%, 17.5%, 1.1%, 4.6% and 48.8%, respectively for severe DDI, and 47.1%, 62.7%, 7.7%, 13.1% and 38.4% for moderate DDI. Without consideration of settings, the distribution was normal (*p* < 0.001) for severe DDI; and a significant difference between the tools (*p* = 0.018) in their ability to detect severe DDIs was found. Concerning moderate DDI, there was a normal distribution (*p* = 0.057) but no significant difference between the tools (*p* = 0.077).

Since Micromedex was the most used tool in the general population (*n* = 14, 20%), we made a comparison of DDI (severe and moderate) according to Micromedex between the settings. The prevalence of severe DDI was 6.1% (95% CI: 4.5–7.7), 2.5% (95% CI: 1.1–3.8) and 32.8% (95% CI: 23.0–42.7) in primary care, nursing home and hospital revealing differences between settings (*p* < 0.001). Concerning moderate DDI, a comparison between the three settings was not possible given that Micromedex was not used to identify such DDI in primary care. However, no difference between nursing home and hospital was found (*p* = 0.057). Indeed, moderate DDI prevalence was 48.3% (95% CI −23.9–120.6) and 49% (95% CI 47.6–50.4) for these settings.

**Figure 1:**
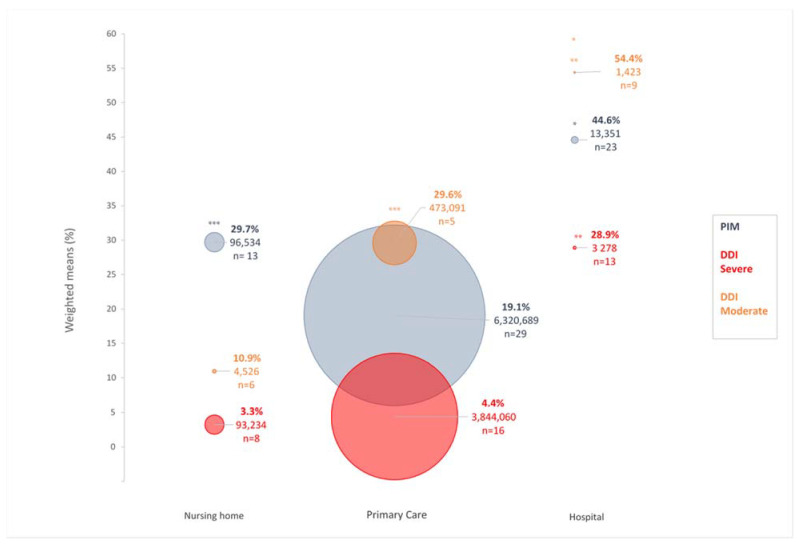
Bubble plot of the prevalence (%) of potentially inappropriate medication (PIM) (grey) and of severe (red) and moderate (orange) drug–drug interaction (DDI) in elderly patients in primary care, nursing home and hospital settings. The prevalence of both PIM and DDI has been reported as a weighed mean. The surface of the circle is proportional to the total sample size of the studies used in the calculation of the parameters (the sample size, the weighed mean and the number of studies used are reported). *: significant difference between hospital and primary care settings. **: significant difference between hospital and nursing home settings. ***: significant difference between primary care and nursing home settings.

The weighted mean prevalence of severe DDI (Figure 1) was in the rank order: hospital 28.9% (95% CI: 17.2–40.6), primary care 4.4% (95% CI 3.2–5.6) and nursing home 3.3% (95% CI: 3.1–3.4). The same rank order was found for moderate DDI with 54.4% (95% CI 38.8–70.0) in hospital, 29.6% (95% CI: 28.5–30.6) in primary care and 10.9% (95% CI 2.3–19.6) in nursing home, respectively.

Severe and moderate DDI displayed significant differences between settings (*p* < 0.001 for each). Using pairwise comparisons, no significant differences were noticed for severe DDI between nursing home and primary care (*p* = 0.21) nor between primary care and hospital (*p* = 0.054). However, the prevalence of severe and moderate DDI between nursing home and hospital was significantly different (*p* < 0.001 and *p* = 0.0035 respectively). Differences in the prevalence of moderate DDI between primary care and hospital (*p* = 0.0035) and between primary care and nursing home (*p* < 0.001) were also observed.

The prevalence in DDI in specific populations of patients and in the different settings is presented in Table 1, Table 2 and Table 3.

### 3.7. Prevalence of Drug–Drug Interactions in the Clinical Data Warehouse

DDI with the 15 selected pairs (DDI as victim drug and inhibitor) occurred in 769 patients of our sample of 8434 patients leading to a global prevalence of 9.1% (Table 5). The prevalence of DDI for these selected pairs ranged from 0.1% (CYP2D6 inhibition of tramadol metabolism by terbinafine) to 19.4% (P-gp transport inhibition of apixaban by amiodarone).

## 4. Discussion

This systematic review showed that the mean weighted prevalence of PIM was in the following rank order: hospital > nursing home > primary care with significant differences between these settings (Figure 1). The prevalence of severe and moderate risk DDI in hospital settings was higher than those in primary care and nursing home (Figure 1), and there were few studies dealing with DDI involving specifically PIM.

### 4.1. Potentially Inappropriate Medications

The polymedication rate was similar between settings, and cannot be accounted for in the differences in PIM prevalence. When considering only studies using Beers criteria, the prevalence of PIM remained the highest in the hospital setting but prevalence in primary care setting exceeded the one in nursing home setting. The highest prevalence of PIM in the hospital setting may be related to the more severe conditions of patients and to the fact that some PIM are due to drug–disease and drug–syndrome interactions (e.g., heart failure, delirium, late stage of chronic kidney disease). Moreover, as recently suggested, hospital admission per se (as a result of change in patient clinical status and of intensification of healthcare) was shown to be an important driver of PIM independently of polymedication and multimorbidity [124]. 

However, differences in PIM prevalence among settings may be somewhat biased by the tools used that have different criteria of detection. Indeed, only a few medications are common to all the PIM’s criteria, resulting from differences in drug dosage as well as market availability [125]. If Beers criteria were the most used tool in the reported studies, NORGEP and STOPP/START were the two other most used tools. The higher prevalence observed with Beers criteria (38.6%), compared to NORGEP (26.0%) may be related to the fact that NORGEP criteria do not include medications that would be considered inappropriate for specific co-morbidities and hence led to an underestimation of the prevalence. On the other hand, the higher prevalence reported with STOPP/START (60.4%) may result from the fact that this tool identifies underuse and overuse of medications [126]. Hence, the prevalence of PIM may be dependent on the tools used in the studies.

### 4.2. Drug–Drug Interactions

We decided to focus only on clinically relevant DDI (i.e., considered as major and moderate risk) that are highly likely to alter the effectiveness or toxicity of one or more medications. Clinically relevant DDI can lead to measurable patient adverse events taking into account an individual patient’s profile. However, there are very few studies considering DDI resulting in actual harm to the patients. 

Reporting only clinically relevant DDI was made possible by a mapping of the different risk rating classification provided by the checker tools that we performed (Table 4). Besides medication severity classification, differences in DDI checker tools also result from differences in the knowledge databases they are based on. A comparison of three commercial DDI knowledge databases showed significant differences in their numbers of clinical medication pairs and a limited overlap [127]. In a data mining study performed in 10,506 elderly patients’ treated statins, we showed differences in the risk rating, and in the individuals DDI among the different statins used [128].

The higher prevalence of severe and moderate risk DDI in hospital settings (Figure 1) compared to primary care and nursing home is not unlikely given the nature of medication treatments that are prescribed to hospitalized patients as a result of more severe conditions. This higher prevalence in hospital setting was apparently not related to the polymedication rate that was very similar among the different settings. 

In a recent systematic review from studies achieved in hospitalized patients [129], the rate of potential DDI (all risk rating) was estimated as 33%, with a rather large variability (95% CI: 17.3–51.3). Our estimation of clinically relevant DDI in elderly patients hospitalized from general population displayed a higher load of DDI since the prevalence of severe and moderate DDI averaged 28.9% and 54.4%, respectively. 

The fact that a rather high prevalence of clinically relevant DDI occurs in a population with a high prevalence of PIM prescription (around half of the population) is of particular concern. Indeed, DDI can occur with a PIM as the victim medication, and may enhance their inappropriate character by increasing their unfavorable balance of benefits and harm. Indeed, the enzymatic inhibition of the metabolism of a PIM may enhance its systemic exposure and, by the way, may increase either its clinical effects and/or its side effects. However, the clinical impact of the increase in the inappropriate character of PIM depends on the magnitude of the increase in systemic exposure as well as on the therapeutic index of the PIM. PIM generating DDI (i.e., as a perpetrator medication) is another concern to investigate. Even though the DDI will not modify per se the inappropriate character of the PIM, it could potentially have deleterious effects related to the induction or the inhibition of the metabolic pathway and/or transporter involved in the disposition of the medication undergoing DDI (i.e., victim medication). 

This review clearly showed that the issue of DDI occurring specifically with PIM has not yet been of concern. Indeed, only 4 studies out of 91 have considered this issue. In a recent study involving 368 patients in a primary care setting, the association between polypharmacy, PIM use and DDI (called “iatrogenic triad”) was evidenced in around 30% of the patients. DDI and PIM were encountered in 8.2% of the patients [39]. In a study involving 1987 nursing home residents, 6.6% of PIM users were susceptible to major DDI [25]. However, these studies did not report the prevalence of DDI involving specifically PIM.

More interestingly, a study involving 120 nursing home residents showed that potential DDI in PIM occurred in 18.3% of the prescriptions with 60% of these DDI considered as potentially harmful [22]. In a study focusing on the use of benzodiazepine in 744 primary care patients, the prevalence of DDI in benzodiazepines considered as PIM occurred in 7% [68].

Ideally, in elderly patients, PIMs should always be reviewed, and DDI should be always checked. However, with regard to DDI, attention should be paid to those occurring with PIM since they could increase their inappropriate character. Even though this issue should be tackled in priority in the hospital setting, it is also relevant for patients in primary care and nursing homes even if the prevalence of clinically relevant DDI was lower, especially in nursing homes (Figure 1). 

Figure 1 also suggests that studies in the hospital setting involving a higher number of patients are warranted to get a better picture of DDI in PIM in this setting. If it could be difficult to reach the sample sizes reported in primary care studies, the development of prescriptome analytics from clinical data-warehouses should help in reaching this goal [130].

An overview of the future management of the burden of DDI of the healthcare system is beyond the scope of this paper, but we think that attention should be paid to DDI in PIM in elderly people given the epidemiologic transition characterized by the population aging and the emergence of chronic diseases requiring long-term medication treatment with medications classified as PIM [10]. In the hospital setting, DDIs are estimated to contribute to 3–5% of adverse events. They are also a significant cause of hospitalization (either via emergency department visits or via hospital admissions). In a retrospective study on the origin of hospital admissions, adverse drug reactions (ADRs) were rated as a significant cause and around 25% of these ADRs were related to a DDI [131].

Given the paucity of data on DDI occurring specifically with PIM in the literature mentioned above, we have performed during the course of this review a preliminary study by checking 15 DDIs involving 6 PIMs as victim medications involving 8464 elderly patients in our hospital. The global prevalence of clinically relevant DDI (i.e., 9.1%) observed in our sample should be considered with caution because only 15 potential DDIs were checked, and it is thus a real underestimation of the true prevalence. However, this short survey of a subset of 15 DDIs of moderate or major risk regarding only 6 PIMs suggests that a comprehensive investigation of DDI involving PIM should implemented. This could be facilitated by using an automatic DDI identification as we previously set up for a study on DDI with statins [128]. 

Furthermore, considering that some of these DDIs were risk-rated as major (Table 5), a further study would ideally investigate the potential negative outcomes of DDI in PIM on ADR, and their consequences (hospitalization as well as morbidity and mortality).

### 4.3. Strength and Limitations 

This systematic review has several strengths. First, our study included a broad range of studies encompassing the primary care, nursing home and hospital settings. Second, given the fact that different DDI checker software are used worldwide with different classifications of risk ratings, we have performed a mapping of the DDI risk rating to allow a better classification of clinically significant DDIs, and of the estimation of their prevalence. Third, this is the first review of DDIs in elderly patients in whom PIMs are prescribed. 

This study has several limitations. First, given the heterogeneity of the selected studies in terms of methodology and tools used as well as in reporting data, there are some missing data precluding a formal meta-analysis and its statistical evaluation. Nevertheless, weighted means in PIM and DDI prevalence allowed us to report some differences between settings. Second, we only performed the literature search using PubMed and Web-of-Science and did not identify grey literature so that some studies may have not been included. Third, the low number of studies in certain combinations (setting/PIM or setting/DDI) may contribute to some uncertainty in the point estimates and 95% CI. Fourth, the huge difference in the total sample size between settings (primary care >> nursing home > hospital) may constitute a bias in comparison.

## 5. Conclusions: Future Research

The current paper presents an in-depth review of the literature regarding the topic of DDI in elderly patients using PIM. It was performed with a mapping of DDI checker tools we thought to be a prerequisite for meaningful comparisons between studies. To our knowledge, this is the first systematic review of this issue.

We showed a higher prevalence of PIM, and of severe and moderate DDI, in the hospital setting compared to nursing home and primary care, unrelated to polymedication rate. Within the studies on DDI in elderly in whom PIM were prescribed, the issue of DDI occurring specifically with PIM has not yet been of concern, since only four studies involving a rather limited number of patients investigated this topic. 

To help clinicians and pharmacists to identify at risk elderly patients, strategies to assess the appropriateness of medication use in elderly should additionally consider DDIs that specifically involve PIM. DDI software providers should implement alerts when a DDI occurs with PIM with a clinical decision support so that healthcare professionals can easily prevent and manage these DDIs. This would be most helpful in the hospital setting where PIMs and DDIs are highly prevalent.

## Figures and Tables

**Table 1:** Prevalence (%) of potentially inappropriate medications (PIM) and drug–drug interactions (DDI) (total, severe and moderate) and polymedication (mean or median) in elderly patients in nursing home setting. At the end of the table, the prevalence of PIM and DDI is reported as the weighted mean (in bold). Footnote: Prospective (P), retrospective (R), mean (M), median (m), threshold (t), severe (S), moderate (M), not apply (NA), not determined (ND).

	Author	Year	Country	Age (Mean, Median or Treshold)	Sample Size	Study Style P–R	Source of Data (Medical Chart–Database)	PIM Criteria	Prevalence of PIM (% Patients with at least 1 PIM)	DDI Checkers (Access Date or Version)	Prevalence of DDI (% of Patients)				Polymedication (Mean or Median Number of Medications)
											All Risk Rating	Severe and Moderate Risk Rating	Severe	Moderate	
*1*	Bitter K et al. [15]	2019	Germany	84	103	R	Medical chart	PRISCUS	16	ABDA (2018)		40	ND	ND	13
*2*	Kolar J et al. [16]	2018	Slovak Republic	79.9	70	R	Medical chart	Topinkova list (2012)	34.3	Lexicomp (2016)	88.4		8.2	100	7.87
*3*	Fog AF et al. [17]	2017	Norway	85.9	2465	P	Medical chart	NORGEP, STOPP/START	1.2	DRUID (2017)	4.4		ND	ND	9.8
*4*	Alves-Conceição V et al. [18]	2017	Brazil	81.8	125	R	Medical chart	Beers Criteria (2012)	73.6	Micromedex (2014)	62.4		ND	ND	ND
*5*	Lao CK et al. [19]	2013	Macao	86.6	114	R	Medical chart	STOPP/START	46.5	Micromedex (2012), Lexicomp (2012)	37.8		ND	ND	6.9
*6*	Xavier-Pinto MC et al. [20]	2013	Brazil	76.7	151	R	Medical chart	Beers Criteria (2012)	25.8	Micromedex (2012)	54.1		3.1	81.4	3.3
*7*	Bakken MS et al. [21]	2012	Norway	84.7	157	P	Medical chart	NORGEP	25.5	DRUID (2011)	52.9		1.3	6.4	6.0
*8*	Varallo FR et al. [22]	2012	Brazil	>60 (t)	120	R	Medical chart	Fick criteria (2003) (updated Beers Criteria), WHO criteria	29.2	Micromedex (2011)		8.3	1.7	6.7	ND
*9*	Haassum Y et al. [23]	2012	Sweden	85.6	86721	R	Database (Swedish Prescribed Drug Register (SPDR))	Swedish National Board of Health and Welfare	30.2	Swedish Physicians’ Desk Reference (2010)	ND	ND	3.3		7.2
*10*	Halvorsen KH et al. [24]	2012	Norway	85.3	2986	R	Database (Norwegian prescription database)	NORGEP	31.4	DRUID (2011)	48		1	6	5.7
*11*	Hosia-Randell HMV et al. [25]	2008	Finland	83.7	1987	R	Medical chart	Fick criteria 2003 (updated Beers Criteria)	34.9	Swedish, Finnish, Interaction X-referencing (SFINX) (2007)	ND	ND	4.8		7.9
*12*	Nygaard HA et al. [26]	2003	Norway	86.3	1042	R	Medical chart	Beers Criteria (1997)	12.8	Norwegian Pharmaceutical Products Compendium (2000)	ND	10.4	3.0	10.1	5
*13*	Giron MST et al. [27]	2001	Sweden	>81 (t)	493	P	Medical chart	Swedish National Formulary	80.1	Swedish Physicians’ Desk Reference (1998)	ND	24.5	ND	ND	4.5
					96,534				**29.7**		**31.1**	**15.9**	**3.3**	**10.9**	
14	Stuhec M et al. [28]	2019	Slovenia	80.6	24 Mental health disease	P	Medical chart	Beers criteria (2015), PRISCUS	70.8	Lexicomp (2019)	ND	ND	50	ND	12.2
15	Bazargan M et al. [29]	2019	USA	75.2	193 Hypertension	P	Medical chart (drug containers)	Beers criteria (2015)	46	Beers criteria (2015)	NA	23	ND	ND	7,3
16	Allegri N et al. [30]	2017	Italy	80.4	860 Mental health disease	R	Medical chart	Beers criteria (2015)	44.4	Drugs.com (2016)	ND	30.9	ND	ND	5.9
17	Pasina L et al. [31]	2016	Italy	84.9	272 Mental health disease	P	Medical chart	(2012) Beers criteria, STOPP/START	74.6 (psychotropic drugs)	INTERcheck (2013)	86.4	53.3	ND	ND	7
18	Hanlon JT et al. [32]	2015	USA	>65	1076 Mental health disease (mild to moderate)	R	Database (Medication dispensing information from the Pharmacy Benefits Management services (PBM))	Beers criteria (2012)	27.2	VA PBM and Medical Advisory Panel Cholinesterase inhibitor criteria for use (2003)		7.9			
					227 Mental health disease (severe)				25.1			5.3			
19	Hanlon JT et al. [33]	2011	USA	>65	877 Mental health disease	P	Database (Medication dispensing information from the Pharmacy Benefits Management services (PBM))	AMDA Guideline, VHA/DOD guideline	41.3	AMDA Guideline (2003), VHA/DOD guideline (2010)	ND	25.9			
					2815 Control				96		ND	ND			
20	Giron MS et al. [27]	2001	Sweden	>81	188 Mental health disease	P	Medical chart	Swedish National Formulary	82.9	Swedish Physicians’ Desk Reference (1998)	ND	26.6			4.6
					**6532**				**65.2**		**86.4**	**22.5**	**50**	**NA**	

**Table 2:** Prevalence (%) of PIM and DDI (total, severe and moderate) and polymedication (mean or median) in elderly patients primary care setting. At the end of the table, the prevalence of PIM and of DDI is reported as the weighted mean (in bold). Footnote: Prospective (P), retrospective (R), mean (M), median (m), threshold (t), severe (S), moderate (M), not apply (NA), not determined (ND).

	Author	Year	Country	Age (Mean, Median or Treshold)	Sample Size	Study Style P–R	Source of Data (Medical Chart–Database)	PIM Criteria	Prevalence of PIM (% Patients with at least 1 PIM)	DDI Checkers (Access Date or Version)	Prevalence of DDI (% of Patients)				Polymedication (Mean or Median Number of Medications)
											All Risk Rating	Severe and Moderate Risk Rating	Severe	Moderate	
*1*	Bobrova et al. [34]	2019	Finland	84.6	208	R	Database (PharmaService’s documentation system ANJA)	EU [7], PIM list	73	INXBASE (2017)	ND	ND	2.4	50	5.9
*2*	Vatcharavongvan et al. [35]	2019	Thailand	70.5 (m)	400	R	Medical chart	Beers Criteria (2015), STOPP/START, Winit–Watjana	75.3	2015 Beers criteria	NA	16	NA	NA	11(m)
								Beers Criteria (2015)	59						
								STOPP/START	40.3						
								Winit–Watjana	66.8						
*3*	Toivo et al. [36]	2019	Finland	81.6	65	P	Medical chart	Beers criteria (2015)	93.9	SFINX (2018)	ND	ND	10.8	ND	10.4
				84.0	64				90.6		ND	ND	1.6	ND	9.8
*4*	Stuhec et al. [37]	2019	Slovenia	77.5	91	R	Medical chart	PRISCUS	ND	Lexicomp (version 4.0.1 and 4.0.2)	ND	ND	54.9	ND	13.8
*5*	Patel R et al. [38]	2018	USA	>65 (t)	703	P	Medical chart	Beers criteria (2015)	29	Beers criteria (2015)	NA	7.7	NA	NA	5.7
*6*	Novaes PH et al. [39]	2017	Brazil	73.8 (>60)	368	R	Medical chart	Beers criteria (2012)	42.1	Medscape Drug Interaction Checker (2016)	72.3	ND	17.9	ND	4.46
								STOPP (2015)	46.2						
*7*	Hanlon JT et al. [40]	2017	USA	73.6	3055	R	Database (Health ABC study)	Beers criteria (2015)	34.0	Panel of 70 DDI used for the study (2017)	ND	25.1	ND	ND	NA
*8*	Marín-Gorricho R et al. [41]	2017	Spain	85.2	103	R	Medical chart	STOPP/START (2014)	81.6	CheckTheMeds (2017)	57.3	ND	ND	ND	7.4
*9*	Bazargan M et al. [42]	2016	USA	73.5	400	R	Interview	Beers criteria (2012)	69.5	Healthline drug interaction checker (2015)	ND	ND	52.75	ND	NA
*10*	Popovic B et al. [43]	2014	Croatia	77	29418	R	Database (Croatian Health Insurance Fund database)	Mimica Matanović and Vlahović-Palčevski (2012)	62.4	Panel of 49 potentially serious DDI used for the study (2014)	ND	ND	ND	ND	NA
*11*	Tora H et al. [44]	2014	Sweden	75.8	180059	R	Database (Swedish national prescription repository)	Swedish National Board of Health and Welfare	30.8	First Databank’s DDI module (2013)	ND	38.6	ND	ND	10.4
*12*	Steinman MA et al. [45]	2014	USA	75	462405	R	Database (Merged VA database-Medicare Program)	Beers criteria (2012)	26.0	Lexicomp (2013)	ND	30.2	4.4	30	NA
*13*	Lund BC et al. [46]	2013	USA	75.8	1549824	R	Database (VA database)	Zhan criteria (adapted from 1997 Beers criteria)	17.9	Hansten PD, Horn JR. Applied Therapeutics Inc. (1998)	NA	3.75	NA	NA	5.5
								Fick criteria 2003 (updated Beers Criteria)	16.5						
*14*	Koper D et al. [47]	2013	Austria	76.4	169	P	medical chart	PRISCUS	37.3	Lexicomp (2012)	ND	59.2	2.4	58	9.1
*15*	Haasum Y et al. [23]	2012	Sweden	75.6	1260843	R	Database (Swedish Prescribed Drug Register SPDR)	Swedish National Board of Health and Welfare	11.6	Swedish Physicians’ Desk Reference (2010)	ND	ND	3.2	ND	4.3
*16*	Halvorsen KH et al. [24]	2011	Norway	83.0	8268	R	Database (Norwegian prescription database)	NORGEP	24.6	DRUID (2011)	57	ND	2	10	5.7
*17*	Ghadimi H et al. [48]	2011	Iran	73.0	2041	R	Medical chart	Fick criteria 2003 (updated Beers Criteria)	30.3	Swedish Physicians’ Desk Reference (2009)	ND	14.3	2.4	13.1	4.4
*18*	Pozzi C et al. [49]	2010	Italy	73.0	1022	R	Database (ICARe Dicomano Study database)	Beers Criteria (1991)	9.0	Micromedex (2009)	ND	ND	7.2	ND	2.2
*19*	Johnell K et al. [50]	2009	Sweden	81.0	645429	R	Database (Swedish Prescribed Drug Register SPDR)	Swedish NationalBoard of Health and Welfare	22.5	Swedish Physicians’ Desk Reference (2009)	ND	ND	6.9	ND	6.8
*20*	Chrischilles EA et al. [51]	2009	USA	>68 (t)	626	P	Database (Iowa medicare beneficiaries)	Beers Criteria (1997)	51.4	Hansten PD, Horn JR. Applied Therapeutics Inc. (1998)	NA	5.8	NA	NA	8.5
*21*	Haider SI et al. [52]	2009	Sweden	80.9	626258	R	Database (Swedish Prescribed Drug Register SPDR)	Swedish National Board of Health and Welfare	34.6	Swedish Physicians Desk Reference (2007)	ND	25	ND	ND	5.8
*22*	Lapi F et al. [53]	2009	Italy	72.7	568	R	Database (ICARe Dicomano study database)	Beers Criteria (1991)	9.1	Micromedex (2008)	ND	20.1	4.7	ND	NA
				77.7					5.1		ND	30.5	5.6	ND	NA
*23*	Johnell K et al. [54]	2008	Sweden	>75 (t)	122413	R	Database (Swedish Prescribed Drug Register SPDR)	Swedish National Board of Health and Welfare	40.3	Swedish Physicians’ Desk Reference (2007)	ND	ND	8.8	ND	9.4
					606692				13.6		ND	ND	3.7	ND	4.6
*24*	Johnell K et al. [55]	2007	Sweden	82	732228	R	Database (Swedish Prescribed Drug Register SPDR)	Swedish National Board of Health and Welfare	16.5	Swedish Physicians’ Desk Reference (2007)	ND	ND	4.1	ND	5.4
*25*	Bregnhøj L et al. [56]	2007	Denmark	76.6	212	R	Database (Drug subsidy system of Danish pharmacies)	MAI	84	Hansten and Horn (2004)	NA	0.6	NA	NA	7.6
*26*	Cannon KT et al. [57]	2006	USA	78	786	R	Medical chart	Beers criteria (1997)	31	Multidisciplinary Medication Management (M3) Project criteria (2005)	NA	NA	10	NA	8.0
*27*	Zhan C et al. [58]	2005	USA	> 65 (t)	70203	R	Database (National Ambulatory Medical Care Survey NAMCS)	1997 Beers criteria, Mac Leod 1997, Panel expert 50 drug-disease combinations	2.58	1997 Beers criteria, Mac Leod 1997, Panel expert 6 drug-drug combinations	NA	0.76	NA	NA	NA
*28*	Azoulay L et al. [59]	2003	Iran	72.6	3000	R	Medical chart	Beers criteria (1997)	27.6	Micromedex (Drug–Reax vol. 114)	9.5	ND	ND	ND	3.7
*29*	Tamblyn R et al. [60]	2003	Canada	75.4	6284	P	Medical chart	McLeod 1997	31.8	McLeod (1997)	NA	2.6	NA	NA	NA
				75.3	6276				33.3		NA	2.4	NA	NA	NA
*30*	Schmader K et al. [61]	1994	USA	69.8	208	R	Medical chart	MAI	14	MAI (1994)	NA	0	NA	NA	NA
					**7,872,649**				**19.1**		**45.3**	**14.65**	**4.4**	**29.6**	
31	Courlet P [62]	2019	Switzerland	71	122 HIV	P	Database (Swiss HIV cohort study)	Beers criteria (2015)	31	University of Liverpool drug interaction checker, Up-to-Date database (2017)	11	ND	ND	ND	4
32	Lund BC et al. [63]	2017	USA	>66	19318 Cancer	R	Database (SEER Medicare)	Beers criteria (2012)	37.3	Micromedex (2017)			5	3.2	
33	Oesterhus R et al. [64]	2017	Norway	77	251 Mental health disease	R	Database (DemWest study)	NORGEP	14	Norwegian drug interaction database (2014, Norwegian medicines agency)	41		1.6	36	4
34	Wucherer D et al. [65]	2017	Germany	79.8	446 Mental health disease	R	Database (Dementia DelpHi–MV study)	PRISCUS	22.9	ABDA (2017)	ND	ND	3.8	34.8	6.4
35	Yazdanshenas H et al. [66]	2016	USA	>65	187 Pain	R	Medical chart	Beers criteria (2012)	83	Healthline (2015)	60	ND	ND	ND	
36	Suehs B.T. et al. [67]	2016	USA	>65	66275 Overactive bladder	R	Medical chart	Beers criteria (2012)	31.1	Beers criteria (2012)		14.3 (anticholinergic DDI)			
37	Dionne PA et al. [68]	2013	Canada	73.8	744 Overactive bladder	R	Database (ESA survey)	Fick criteria 2003 (updated Beers Criteria)	22	Micromedex (2012)	15	ND	ND	ND	
38	Gallagher [69]	2001	USA	88	146 Heart Failure	R	Medical chart	Beers criteria (1997)	8.2	Panel of DDI used for the study (1999)	ND	44.5	ND	ND	8
					**87,489**				**32.4**		**26.1**	**14.4**	**4.9**	**4.3**	

**Table 3:** Prevalence (%) of PIM and DDI (total, severe and moderate) and polymedication (mean or median) in elderly patients in hospital setting. At the end of the table, the prevalence of PIM and of DDI is reported as the weighted mean (in bold). Footnote: Prospective (P), retrospective (R), mean (M), median (m), threshold (t), severe (S), moderate (M), not apply (NA), not determined (ND).

	Author	Year	Country	Age (Mean, Median or Treshold)	Sample Size	Study Style P–R	Source of Data (Medical Chart—Database)	PIM Criteria	Prevalence of PIM (% Patients with at least 1 PIM)	DDI Checkers (Access Date or Version)	Prevalence of DDI (% of Patients)				Polymedication (Mean or Median Number of Medications)
											All Risk Rating	Severe and Moderate Risk Rating	Severe	Moderate	
1	Al-Azayzih A et al. [70]	2019	Jordan	73.2	4622	R	Medical chart	Beers criteria (2015)	62.5	Beers criteria (2015)	NA	1.6	NA	NA	NA
2	Ertuna E et al. [71]	2019	Turkey	80.3	91	R	Medical chart	Beers criteria (2015), STOPP/START	24.17	RxMediaPharma Interactive Drug Database (2018)	40.12		ND	ND	8.17
3	De Oliveira S.B.V. et al. [72]	2018	Brazil	76 (m) > 60 yrs	170	P	Medical chart	Beers criteria (2015)	44.7	Micromedex (2017)	55.3		ND	ND	11 (m)
4	Colombo F. et al. [73]	2018	Italy	77 (m)	122	P	Medical chart	EU [7]–PIM list	69.6	Micromedex (2017)	78.7		26,1	50,1	7 (m)
								Beers Criteria (2015)	72,6						
5	Ruiz-Millo O. et al. [74]	2018	Spain	81.1	162	P	Medical chart	STOPP/START	90.7	Micromedex (2017)		80,2	51,9	48,1	12.2
6	Saqib A et al. [75]	2018	Pakistan	> 60 (t)	432	R	Medical chart	Beers criteria (2015)	48.3	Drugs.com interaction checker (2017), Stockley’s drug interaction checker (2017)	61.8		6.9	74,3	4.74
7	Gutiérrez-Valencia M et al. [76]	2017	Spain	88.3	200	R	Medical chart	Beers criteria (2012)	68.5	BOT Plus tool (2016)	82.5		24	66.5	9.1
								2015 STOPP	71						
8	Franchi C et al. [77]	2016	Italy	83.7	347	P	Medical chart	Beers criteria (2012), START/STOPP, MAI, IPET	39.1	INTERcheck (2013)	91.4		58.3		5.7
				83.8	350				44.7		85.6		53.9		6.3
9	De Melo DL et al. [78]	2016	Brazil	73.7 (>60)	316	P	Medical chart	Beers criteria (2015)	85.8	Micromedex (2014)	44.5		19.9		5.73
10	Grion AM et al. [79]	2016	Italy	86.5	100	P	Medical chart	STOPP/START	53	Micromedex (version 2.0)			38		6
					449				54				37		6
11	Salwe K et al. [80]	2016	India	71.6	100	R	Medical chart	Beers criteria (2012)	6.3	Drugs.com interaction checker (2013)	52.7		3.7	40,4	7.6
12	Matanovic SM et al. [81]	2014	Croatia	>65 (t)	454	R	Medical chart	Beers criteria (2012)	57.9	Matanović & Vlahović-Palčevski self screening tool (2014)	32.8		ND	ND	5.3
								Suzana Matanović & Vlahović-Palčevski self screening tool	44.1						
13	Franchi C et al. [82]	2014	Italy	82.8	39	P	Medical chart	Beers criteria (2012)	25.6	Italian Formulary Guida all uso dei farmaci (2013)	56.4		12.8	51.3	6 (m)
				81.6	39				17.95		76.9		15.4	66.7	7 (m)
14	Ghibelli S et al. [83]	2013	Italy	81.3	74	P	Medical chart	Beers criteria (2012)	39.1	INTERcheck (2013)	90.5	86.5	37,8	48,6	NA
				81.1	60				41.7		85	73.3	45	28,3	NA
15	Bakken MS et al. [21]	2012	Norway	84.7	133	P	Medical chart	NORGEP	22.6	DRUID (2011)	54.9		0	8.3	6.0
16	Siqueira JS et al. [84]	2012	Brazil	77.5 (m)	62	R	Medical chart	Fick criteria 2003 (updated Beers Criteria)	34	Micromedex (2011)	77		23,9	49,3	11
17	Trivalle C et al. [85]	2010	France	83.6	526	P	Medical chart	Beers criteria (1997)	13	Vidal (2005)	6		ND	ND	9.4
18	Schuler J et al. [86]	2008	Austria	>75 (t)	543	R	Medical chart	Beers criteria (1997)	30.1	Medis Software (2008)		65.8			7.5
19	Nixdorff N et al. [87]	2008	USA	75	124	P	Medical chart	Fick criteria 2003 (updated Beers Criteria)	34.6	Lexicomp (2007)	26.6		ND	ND	8.6
20	Radosevic N et al. [88]	2008	Croatia	65.9	225	R	Medical chart	Fick criteria 2003 (updated Beers Criteria)	25	Fick criteria 2003 (updated Beers Criteria)	NA	22.2	NA	NA	5.78
21	Saltvedt I et al. [89]	2005	Norway	81.8	127	R	Medical chart	Beers criteria (1997)	10	DRUID (1999), Hansten PD, Horn JR. Applied Therapeutics Inc. (2000)	44		0	ND	4 (m)
				82.4	127				9		52		0	ND	4 (m)
22	Fillenbaum GG et al. [90]	2004	USA	> 65(t)	3237	R	Database (Established Populations for Epidemiologic Studies of the Elederly (EPESE))	Beers criteria (1997)	21.3	Beers criteria (1997)	NA	13.3	NA	NA	NA
23	Frank C et al. [91]	2001	Canada	77.8	120	R	Medical chart	Beers criteria (1997)	7.4	Clinidata Drug Interactions program (1996)	NA	10.6	NA	NA	NA
					**14,127**				**44.6**		**53.8**	**12.9**	**26.9**	**54.4**	
24	Jorgensen TL [92]	2020	Denmark	>70 (t)	1294 Cancer	R	Database (Danish Gynaecological Cancer Database (DGCD))	EU [7]–PIM list (PIM A)	48.6	Micromedex (2019), Lexicomp (2019)	ND	38.6	ND	ND	
								EU [7]–PIM list (PIM B)	33.8						
25	Hong S et al. [93]	2020	South Korea	75	301 Cancer	P	Database (Korean Cancer Study Group PC13-09)	2015 Beers criteria	45.5	Lexicomp (2019)		30.6	1,5	28,5	4.7
26	Lavan A [94]	2019	Ireland	72.5	186 Cancer	P	Medical chart	STOPP/START	73.1	Stockley’s interaction checker (2019)			50.5	ND	7
27	Graf J et al. [95]	2018	Germany	71	1508 COPD	R	Database (COPD cohort COSYCONET)	PRISCUS	10.2	AiD-Klinik system Heidelberg University Medical Center (2017)			4.3	6.4	5
28	Leger DY et al. [96]	2018	France	81.5	122 Cancer	P	Medical chart	Laroche	34.4	Theriaque (2016), e-Vidal (2016)	71.3		3.3	13.9	6.6
29	Alkan A et al. [97]	2017	Turkey	70	159 Cancer inpatients	R	Medical chart	2012 Beers Criteria	48.4	Lexicomp (2016)		47.2			
					286 Cancer outpatients				14.2			28.3			
30	Rougé Bugat ME et al. [98]	2017	France	83.3	106 Cancer	R	Medical chart	Laroche, START tool	61.3	Panel of DDI used for the study (2016)	16		ND	ND	6.67
31	Vrijkorte E et al. [99]	2017	Netherlands	> 65 (t)	60 Cancer	P	Medical chart	Oncostrip	78	Oncostrip (2016)	12		ND	ND	ND
32	Parian A et al. [100]	2015	USA	70	190 Inflammatory Bowel Disease patients	R	Medical chart	2012 Beers Criteria	35	Micromedex (2014)	73.7		ND	ND	6
33	Jarchow AA & Mangoni AA [101]	2013	UK	84	148 Gastric diseases	R	Medical chart	NICE, British National Formulary, SIGN	99.3	British National Formulary (2009)	56.5		ND	ND	7
					**5654**				**35.0**		**53.5**	**37.4**	**7.9**	**10.3**	

**Table 4:** Mapping of the potential risk rating classification of DDI among checker tools used in the studies referenced in the current systematic review.

DDI Checker Tool	Risk Rating Used for the Current Systematic Review	Risk Rating Reported by the DDI Checker Tools	Description
ABDA database [102]	Major	Serious	Life threatening, permanent physical disabilities are probable
	Moderate	Moderate	Dosage adaptation is necessary and/or concomitant treatment requires continuous monitoring.
		Minor	DDI is barely affecting patient’s health. DDI applies for special patients groups
		Insignificant	
		No evidence	
AiD system Heidelberg University Medical Center [95]	Major	Red	Clinically serious
	Moderate	Orange	Potentially clinically relevant
Beers criteria [10]	Major	Major	Potentially Clinically Important Drug–Drug Interactions That Should Be Avoided in Older Adults
BOT Plus tool [103]	Major	Potentially serious	High risk: interactions that are normally serious or frequent, and/or that it is generally advisable to avoid the association
	Moderate	Moderate	Less serious and/or frequent interactions, fundamental recommendation is to administer both active ingredients together with caution, monitoring the patient.
		Mild	Mild and normally rare interactions and/or interactions that have not been recorded in clinical practice, but due to the pharmacology of the active ingredients or the existence of interaction between similar active ingredients, it can be assumed that they could occur on some occasions.
CheckTheMeds [104]	Major	Contraindication	Avoid association
		To avoid	Must be decided or not to modify the treatment, consider an action to minimize the interaction
	Moderate	To be considered	
Clinidata Drug Interaction Program [91]	Major	Severe	
	Moderate	Moderate	
		Mild	
Drugs.com Interaction Checker software [105]	Major	Major	
	Moderate	Moderate	
		Minor	
Norwegian drug interaction database (DRUID) [17]	Major	Major	Drug combinations to avoid
	Moderate	Moderate	May be combined, but precautions need to be taken, e.g., dose changes or monitoring of clinical and/or laboratory parameters
		Minor	Only a theoretical chance of a DDI, and drugs may be combined
First Databank’s DDI module [106]	Major	1	Contraindicated drug combination which generally should not be dispensed or administered to the same patient
		2	Action is required to reduce risk of severe adverse interaction
	Moderate	3	Assess risk to patient and take action as needed
Hansten PD and Horn JR—Applied Therapeutics [107]	Major	1	Contraindicated (drug combinations should be avoided)
	Moderate	2	Medication combinations that should be generally avoided.
Hansten—ORCA 2001 [108]	Major	Contraindicated	No situations have been identified where the benefit of the combination outweighs the risk.
		Provisionally contraindicated	The combination increases the risk of adverse effects. Avoid concurrent use unless interaction is desired or no alternative is available. If the combination is used, increased monitoring may be necessary
	Moderate	Conditional	Risk may be increased, depending on the clinical situation. Assess risk and take action as needed.
		Minimal risk	Risk may be increased, depending on the clinical situation. Assess risk and take action as needed.
		No interaction	Evidence suggests that drugs do not interact.
Healthline.com drug interaction checker [109]	Major	Severe	
	Moderate	Moderate	
		Mild	
INTERcheck software [83]	Major	D	Contraindicated (drug combinations should be avoided)
		C	Major DDIs (drug combinations requiring close monitoring for potentially serious clinical consequences, such as severe adverse effects or lack of clinical efficacy)
	Moderate	B	Moderate (drug combinations requiring dose adjustment and/or drug concentration monitoring);
		A	Minor (drug combinations with no known clinical relevance)
INXBASE (formerly SFINX)			
Italian Formulary Guida all uso dei farmaci [110]	Major	Major	
	Moderate	Moderate	
Lexicomp (Lexi-interact) [111]	Major	X	Avoid combination. Data demonstrate that the specified agents may interact with each other in a clinically significant manner. The risks associated with concomitant use of these agents usually outweigh the benefits. These agents are generally considered contraindicated.
		D	Modify regimen. Data demonstrate that the two medications may interact with each other in a clinically significant manner. A patient-specific assessment must be conducted to determine whether the benefits of concomitant therapy outweigh the risks. Specific actions must be taken in order to realize the benefits and/or minimize the toxicity resulting from concomitant use of the agents. These actions may include aggressive monitoring, empiric dosage changes, choosing alternative agents.
	Moderate	C	Monitor therapy. Data demonstrate that the specified agents may interact with each other in a clinically significant manner. The benefits of concomitant use of these two medications usually outweigh the risks. An appropriate monitoring plan should be implemented to identify potential negative effects. Dosage adjustments of one or both agents may be needed in a minority of patients.
		B	No action needed
		A	No interaction
Medscape Drug Interaction Checker software [112]	Major	Contraindicated	
		Serious	Risk of life threatening drug interaction; use alternative drug
	Moderate	Significant	Potential for dangerous interaction, use with caution and monitor closely)
		Minor	Non-significant interaction
Mefis Software (based ABDA database): c.f. ABDA database			
Micromedex (Drug-REAX System) [113]	Major	Contraindicated	The drugs are contraindicated for concurrent use
		Major	The interaction may be life-threatening and/or require medical intervention to minimize or prevent serious adverse effects.
	Moderate	Moderate	The interaction may result in exacerbation of the patient’s condition and/or require an alteration in therapy.
		Minor	The interaction would have limited clinical effects. Manifestations may include an increase in the frequency or severity of the side effects but generally would not require a Major alteration in therapy.
		Unknown	Unknown
MIMS drug alert [114]	Major	Potentially severe	The interaction between these medications may be life-threatening or may cause permanent damage. These medications are not usually used concurrently; medical intervention may be required.
	Moderate	Moderate	These medications may interact resulting in the potential deterioration of the patient’s condition. The patient should be monitored for the possible manifestations of the interaction. Medical intervention or a change in therapy may be required.
		Minor	Clinical effects of the interaction are limited and may be bothersome but would not usually require a major change to therapy. The patient should be monitored for the possible manifestations of the interaction.
		Caution	The interaction may occur based on the mechanism of action of the co-administered medicines. Be alert for increased or decreased effect, depending on the combination of medicines.
		Not clinically significant	The interaction may occur, but the outcome is not clinically significant.
Multidisciplinary Medication Management (M3) Project criteria [57]	Major	Dangerous	Set of 10 common dangerous drug–drug interactions
Norwegian Pharmaceutical Products Compendium 2000 [115]	Major	A	Potentially serious interactions, drugs that should not be combined
	Moderate	B	Drug combinations that may cause altered effects or side effects that may be managed by individualized dose adaptation
PharmAssist software Vs. [116]	Major	High risk	Clinically significant and potentially life-threatening
	Moderate	Moderate risk	Clinically significant, but unlikely to be life threatening
		Low risk	Unlikely to be clinically significant
RxMediaPharma Interactive Drug Database [117]	Major	High	The interaction between these drugs can be life-threatening or cause permanently damage. These drugs are not usually used together, they require medical intervention. An alternative medicine should be used
	Moderate	Moderate	The clinical impact of interaction is limited, but can be disturbing. Patient should be monitored for the findings of interaction
		Low	Caution should be taken with regard to the reduced or increased efficacy related to the combined drugs
SFINX classification system of DDIs (Swedish, Finnish, Interaction X-referencing), currently INXBASE [118]	Major	D	Clinically relevant DDIs that should be avoided.
	Moderate	C	Clinically relevant DDIs that can be handled with individual dose adjustment, for example
		B	Clinical relevance is unknown and/or varies.
		A	Clinically insignificant DDIs.
Stockley’s interaction checker [119]	Major	Severe	For interactions that could totally incapacitate a patient or result in either a permanent detrimental effect or a life-threatening event.
	Moderate	Moderate	For interactions that could result in an effect that may either cause considerable distress or partially incapacitate a patient. These interactions are unlikely to be life-threatening or result in long-term effects.
		Mild	For interactions that could result in an effect that is mild and unlikely to unduly concern or incapacitate the majority of patients.
		Nothing expected	For interactions that are unlikely to result in an effect, or for drugs pairs where nointeraction occurs
Swedish Physicians’ Desk Reference [50]	Major	D	Clinically relevant DDIs that should be avoided.
	Moderate	C	Clinically relevant DDIs that can be handled with individual dose adjustment, for example
		B	Clinical relevance is unknown and/or varies.
		A	Clinically insignificant DDIs.
Theriaque (based on ANSM Thesaurus) [120]	Major	Contra-indicated	Contraindication is absolute and must not be transgressed
		Should be avoided	The combination advised against should most often be avoided, except after careful consideration of the benefit/risk ratio. It requires close monitoring of the patient.
	Moderate	Precaution of use	The association is possible as soon as simple recommendations to avoid the occurrence of interaction (dosage adjustment, strengthening of clinical, biological monitoring, ECG, etc.) are respected, especially at the start of treatment.
		To take into account	The risk of drug interaction exists. It most often corresponds to an addition of undesirable effects. No practical recommendations can be made. It is up to the doctor to assess the advisability of the association.
University of Liverpool HIV drug interaction checker [121]	Major	Red	Contra-indicated
		Amber	Potentially clinically relevant DDI requiring either dose adaptation or close clinical monitoring
	Moderate	Yellow	Interaction of weak intensity not requiring additional action
		Green	No interaction
Up-to-Date: c.f. Lexicomp			
Vidal (based on ANSM Thesaurus): c.f. Theriaque			

**Table 5:** Prevalence of DDI of moderate or major intensity (by inhibition of a metabolic or of a transport pathway) regarding six selected PIM in hospitalized patients. Data were extracted from the clinical data warehouse of Rennes University Hospital (eHop). DDI were checked by using Micromedex and literature search in Pubmed. Specific bibliographic references were added when DDI was not indexed in Micromedex. Footnote: not indexed (-).

PIM	Patient Sample Size	CYP and/or Transporter Pathway	Inhibitors	Patients with DDI	Prevalence (%)	Risk Rating from Micromedex	Mechanism	Effects
Tramadol	5 896	2D6	Fluoxetine	69	1.17%	Major	Inhibition of CYP2D6-mediated tramadol metabolism	Increased tramadol exposure and reduced concentrations of the active metabolite (risk of signs and symptoms of opioid withdrawal, reduction of efficacy and serotonin syndrome)
			Paroxetine	168	2.85%			
			Terbinafine	6	0.10%			
Apixaban	1 922	3A4/P-gp	Posaconazole	2	0.10%	-	CYP3A4 inhibition	Bleeding risk [122]
			Voriconazole	4	0.21%	-	CYP3A4 inhibition	
			Clarithromycin	20	1.04%	-	CYP3A4 inhibition and P-gp inhibition	
			Verapamil	46	2.39%	-	P-gp competition	
			Amiodarone	366	19.04%	-	P-gp competition	
Digoxin	345	P-gp	Verapamil	11	3.19%	Major	Inhibition of renal and/or extrarenal clearance, additive effects on AV node conduction	Increased serum digoxin concentrations and risk of digitalis toxicity and complete heart block
			Clarithromycin	3	0.87%	Major	Inhibition of P-glycoprotein-mediated digoxin efflux transport by clarithromycin	Increased risk of digoxin toxicity (nausea, vomiting, arrhythmias)
			Amiodarone	60	17.39%	Major	Inhibition of p-glycoprotein by amiodarone, and reduction of digoxin clearance; interference with amiodarone by digoxin	Increased risk of digoxin toxicity (nausea, vomiting, arrhythmias) and potentiated effects of amiodarone (bradycardia, sinus arrest, AV block). Drug interactions may persist for weeks to months after amiodarone discontinuation.
Clozapine	116	1A2	Ciprofloxacin	1	0.86%	Major	Inhibition of CYP1A2-mediated clozapine metabolism and additive QT-interval prolongation	Increased clozapine exposure and risk of QT prolongation
Glimepiride	99	2C9	Amiodarone	10	10.10%	Moderate	Inhibition of CYP2C9-mediated glimepiride metabolism by amiodarone	Increased plasma levels of glimepiride
			Fluconazole	2	2.02%	-	Inhibition of CYP2C9-mediated glimepiride metabolism by amiodarone	Risk of hypoglycemia [123]
Quetiapine	56	3A4	Clarithromycin	1	1.79%	Major	Inhibition of CYP3A4-mediated metabolism of quetiapine; additive effects on QT-interval prolongation	Increased quetiapine exposure and increased risk of QT prolongation

## Data Availability

Not applicable.

## Supplementary Materials

The following are available online at https://www.mdpi.com/1999-4923/13/2/266/s1, Supplementary material 1: PRISMA checklist; Supplementary material 2. PRISMA flow chart of review screening, selection, exclusions and final included studies; Supplementary material 3: Pubmed literature search: Drug interactions [MeSH Terms] AND (((((((((((Inappropriate prescribing[MeSH Terms]) OR Inappropriate Medication[Title/Abstract]) OR Inappropriate Medications[Title/Abstract]) OR Inappropriate Prescription[Title/Abstract]) OR Inappropriate Prescriptions[Title/Abstract]) OR Inappropriate Prescribing[Title/Abstract]) OR Inappropriate Medicines[Title/Abstract]) OR Inappropriate Drugs[Title/Abstract]) OR Inappropriate Drug[Title/Abstract]) OR high-risk medications[Title/Abstract]) OR Inappropriate Use[Title/Abstract]) AND (((((Aged [MeSH Terms OR (Aged, 80 and over[MeSH Terms])) OR elderly[Title/Abstract]) OR older adult[Title/Abstract]) OR older people[Title/Abstract]) Web of Science literature search: TS = (“Drug interactions”) TS = (“Inappropriate prescribing” OR “Inappropriate Medication” OR “Inappropriate Medications” OR “Inappropriate Prescription” OR “Inappropriate Prescriptions” OR “Inappropriate Prescribing” OR “Inappropriate Medicines” OR “Inappropriate Drugs” OR “Inappropriate Drug” OR “high-risk medications” OR “Inappropriate Use”) TS = (Aged OR Aged, 80 and over OR elderly OR older adult OR older people); Supplementary Material 4. Main characteristics of the studies from general population in primary care, nursing home and hospital settings (IQR* = inter-quartile range).
