# Supplementary material for: Drug–Drug Interactions in Elderly Patients with Potentially Inappropriate Medications in Primary Care, Nursing Home and Hospital Settings: A Systematic Review and a Preliminary Study
Source: Pharmaceutics. 2021 Feb 16;13(2):266. doi: 10.3390/pharmaceutics13020266 (PMC7919637; doi:10.3390/pharmaceutics13020266)
Supplement: Supplementary file 1 [file pharmaceutics-13-00266-s001.pdf]

pharmaceutics

Supplementary Materials: Drug–Drug Interactions in Elderly Patients with Potentially Inappropriate Medications in Primary Care, Nursing Home and Hospital Settings: A Systematic Review and a Preliminary Study

Mathilde Bories [1,2,3], Guillaume Bouzillé [2], Marc Cuggia [2] and Pascal Le Corre [1,3,4,*]

Publisher's Note: MDPI stays neutral with regard to jurisdictional claims in published maps and institutional affiliations.

Copyright: © 2020 by the authors. Submitted for possible open access publication under the terms and conditions of the Creative Commons Attribution (CC BY) license (http://creativecommons.org/licenses/by/4.0/).

Supplementary Material 1: PRISMA checklist (2009)

Section/topic	#	Checklist item	Reported on page #
**TITLE**			
Title	1	Identify the report as a systematic review, meta-analysis, or both.	
**ABSTRACT**			
Structured summary	2	Provide a structured summary including, as applicable: background; objectives; data sources; study eligibility criteria, participants, and interventions; study appraisal and synthesis methods; results; limitations; conclusions and implications of key findings; systematic review registration number.	
**INTRODUCTION**			
Rationale	3	Describe the rationale for the review in the context of what is already known.	
Objectives	4	Provide an explicit statement of questions being addressed with reference to participants, interventions, comparisons, outcomes, and study design (PICOS).	
**METHODS**			
Protocol and registration	5	Indicate if a review protocol exists, if and where it can be accessed (e.g., Web address), and, if available, provide registration information including registration number.	

Eligibility criteria	6	Specify study characteristics (e.g., PICOS, length of follow-up) and report characteristics (e.g., years considered, language, publication status) used as criteria for eligibility, giving rationale.	
Information sources	7	Describe all information sources (e.g., databases with dates of coverage, contact with study authors to identify additional studies) in the search and date last searched.	
Search	8	Present full electronic search strategy for at least one database, including any limits used, such that it could be repeated.	
Study selection	9	State the process for selecting studies (i.e., screening, eligibility, included in systematic review, and, if applicable, included in the meta-analysis).	
Data collection process	10	Describe method of data extraction from reports (e.g., piloted forms, independently, in duplicate) and any processes for obtaining and confirming data from investigators.	
Data items	11	List and define all variables for which data were sought (e.g., PICOS, funding sources) and any assumptions and simplifications made.	
Risk of bias in individual studies	12	Describe methods used for assessing risk of bias of individual studies (including specification of whether this was done at the study or outcome level), and how this information is to be used in any data synthesis.	
Summary measures	13	State the principal summary measures (e.g., risk ratio, difference in means).	
Synthesis of results	14	Describe the methods of handling data and combining results of studies, if done, including measures of consistency (e.g., I^2) for each meta-analysis.	
Section/topic	#	Checklist item	Reported on page #
Risk of bias across studies	15	Specify any assessment of risk of bias that may affect the cumulative evidence (e.g., publication bias, selective reporting within studies).	
Additional analyses	16	Describe methods of additional analyses (e.g., sensitivity or subgroup analyses, meta-regression), if done, indicating which were pre-specified.	
**RESULTS**			
Study selection	17	Give numbers of studies screened, assessed for eligibility, and included in the review, with reasons for exclusions at each stage, ideally with a flow diagram.	
Study characteristics	18	For each study, present characteristics for which data were extracted (e.g., study size, PICOS, follow-up period) and provide the citations.	
Risk of bias within studies	19	Present data on risk of bias of each study and, if available, any outcome level assessment (see item 12).	
Results of individual studies	20	For all outcomes considered (benefits or harms), present, for each study: (a) simple summary data for each intervention group (b) effect estimates and confidence intervals, ideally with a forest plot.	

Synthesis of results	21	Present the main results of the review. If meta-analyses are done, include for each, confidence intervals and measures of consistency.	
Risk of bias across studies	22	Present results of any assessment of risk of bias across studies (see Item 15).	
Additional analysis	23	Give results of additional analyses, if done (e.g., sensitivity or subgroup analyses, meta-regression [see Item 16]).	
**DISCUSSION**			
Summary of evidence	24	Summarize the main findings including the strength of evidence for each main outcome; consider their relevance to key groups (e.g., healthcare providers, users, and policy makers).	
Limitations	25	Discuss limitations at study and outcome level (e.g., risk of bias), and at review-level (e.g., incomplete retrieval of identified research, reporting bias).	
Conclusions	26	Provide a general interpretation of the results in the context of other evidence, and implications for future research.	
**FUNDING**			
Funding	27	Describe sources of funding for the systematic review and other support (e.g., supply of data); role of funders for the systematic review.	

Supplementary Material 2: PRISMA flow chart of review screening, selection, exclusions and final included studies

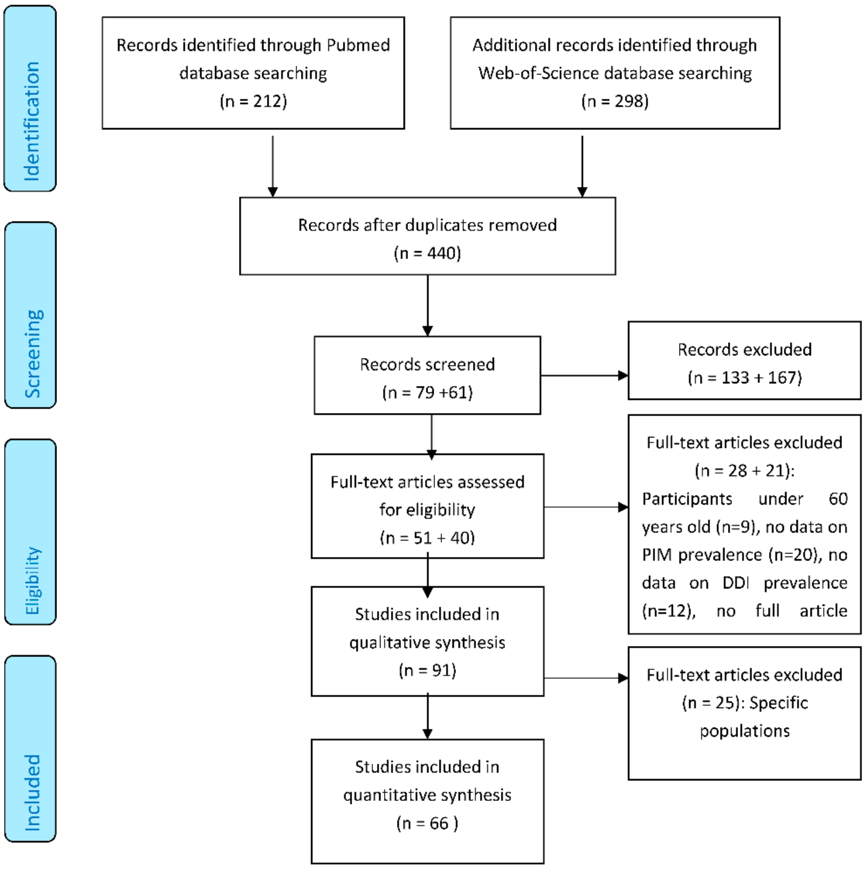

Supplementary material 3

Pubmed literature search :

Drug interactions [MeSH Terms] AND ((((((((((Inappropriate prescribing[MeSH Terms]) OR Inappropriate Medication[Title/Abstract]) OR Inappropriate Medications[Title/Abstract]) OR Inappropriate Prescription[Title/Abstract]) OR Inappropriate Prescriptions[Title/Abstract]) OR Inappropriate Prescribing[Title/Abstract]) OR Inappropriate Medicines[Title/Abstract]) OR Inappropriate Drugs[Title/Abstract]) OR Inappropriate Drug[Title/Abstract]) OR high-risk medications[Title/Abstract]) OR Inappropriate Use[Title/Abstract]) AND (((((Aged [MeSH Terms OR (Aged, 80 and over[MeSH Terms])) OR elderly[Title/Abstract]) OR older adult[Title/Abstract]) OR older people[Title/Abstract])

Web of Science literature search :

TS=("Drug interactions")

TS=("Inappropriate prescribing" OR "Inappropriate Medication" OR "Inappropriate Medications" OR "Inappropriate Prescription" OR "Inappropriate Prescriptions" OR "Inappropriate Prescribing" OR "Inappropriate Medicines" OR "Inappropriate Drugs" OR "Inappropriate Drug" OR "high-risk medications" OR "Inappropriate Use")

TS=(Aged OR Aged, 80 and over OR elderly OR older adult OR older people)

Supplementary Material 4: Main characteristics of the studies from general population in primary care, nursing home and hospital settings (IQR* = inter-quartile range).

	Nursing home	Primary care	Hospital
**Publication year, median (IQR*)**	2012 (2001–2017)	2012 (2008–2017)	2014 (2009–2018)
**Studies, n (%)**	13	30	23
**Countries, n (%)**			
Europe	9 (69.2)	16 (53.3)	13 (56.5)
North America	0 (0.0)	10 (33.3)	3 (13.0)
South America	3 (23.1)	1 (3.3)	3 (13.0)
Asia	1 (7.7)	3 (10.0)	4 (17.4)
**Age, mean (IQR)**	84.7 (82.3–85.8)	76.8 (73–85)	79.5 (76.5–83)
**Sample size, n**	96,534	7,872,649	14,127
**Study style, n (%)**			
Prospective	3 (23.1)	5 (16.7)	11 (47.8)
Retrospective	10 (76.9)	25 (83.3)	12 (52.2)
**Source of data, n (%)**			
Medical chart	11 (84.6)	13 (43.3)	22 (95.7)
Database	2 (15.4)	17 (56.7)	1 (4.3)
**PIM criteria, n (%)**			

Beers Criteria	3 (21.4)	14 (35.9)	17 (56.7)
STOPP/Start	2 (14.3)	4 (10.3)	5 (16.7)
Swedish National Board of Health and Welfare	1 (7.1)	6 (15.4)	0 (0.0)
Fick list	2 (14.3)	2 (5.1)	3 (10.0)
NORGEP	3 (21.4)	1 (2.6)	1 (3.3)
Others	3 (21.4)	12(30.8)	4 (13.3)
**DDI checker, n (%)**			
Micromedex	4 (28.6)	3 (9.7)	6 (25.0)
Swedish Physician's Desk	2 (14.3)	6 (19.3)	0 (0.0)
Lexicomp	2 (14.3)	3 (9.7)	1 (4.2)
DRUID	3 (21.4)	1 (3.2)	2 (8.3)
Intercheck	0 (0.0)	0 (0.0)	2 (8.3)
Others	3 (21.4)	18 (58.1)	13 (54.2)
